# An Overview of the Involvement of D-Serine in Cognitive Impairment in Normal Aging and Dementia

**DOI:** 10.3389/fpsyt.2021.754032

**Published:** 2021-10-11

**Authors:** Magdalena Orzylowski, Esther Fujiwara, Darrell D. Mousseau, Glen B. Baker

**Affiliations:** ^1^Villa Caritas Geriatric Psychiatry Hospital, Edmonton, AB, Canada; ^2^Department of Psychiatry, University of Alberta, Edmonton, AB, Canada; ^3^Neuroscience and Mental Health Institute, University of Alberta, Edmonton, AB, Canada; ^4^Department of Psychiatry, University of Saskatchewan, Saskatoon, SK, Canada

**Keywords:** D-serine, glutamate, NMDA receptor, dementia, Alzheimer's disease, long-term potentiation, aging, cognition

## Abstract

Dementia, of which Alzheimer's disease (AD) is the most common form, is characterized by progressive cognitive deterioration, including profound memory loss, which affects functioning in many aspects of life. Although cognitive deterioration is relatively common in aging and aging is a risk factor for AD, the condition is not necessarily a part of the aging process. The N-methyl-D-aspartate glutamate receptor (NMDAR) and its co-agonist D-serine are currently of great interest as potential important contributors to cognitive function in normal aging and dementia. D-Serine is necessary for activation of the NMDAR and in maintenance of long-term potentiation (LTP) and is involved in brain development, neuronal connectivity, synaptic plasticity and regulation of learning and memory. In this paper, we review evidence, from both preclinical and human studies, on the involvement of D-serine (and the enzymes involved in its metabolism) in regulation of cognition. Potential mechanisms of action of D-serine are discussed in the context of normal aging and in dementia, as is the potential for using D-serine as a potential biomarker and/or therapeutic agent in dementia. Although there is some controversy in the literature, it has been proposed that in normal aging there is decreased expression of serine racemase and decreased levels of D-serine and down-regulation of NMDARs, resulting in impaired synaptic plasticity and deficits in learning and memory. In contrast, in AD there appears to be activation of serine racemase, increased levels of D-serine and overstimulation of NMDARs, resulting in cytotoxicity, synaptic deficits, and dementia.

## Introduction

Dementia, and its most common form, Alzheimer's disease (AD), is a complex and progressive neurological disorder characterized by many neuropsychiatric symptoms, e.g. aggression, anxiety, depression and sleep disorder, and the better known symptoms associated with progressive memory loss and cognitive impairment, all of which can significantly alter the quality of life of those afflicted with this disorder (1, 2). Age is a major risk factor for dementia, and 1.5% of the population will be affected directly by dementia by the age of 65 and >20% of the population by the age of 85 (3). Neurocognitive disorders such as AD are expected to steadily increase in prevalence and incidence as the population ages. It is estimated that the global number of individuals suffering from dementia will reach 65 million by 2030 and 113 million by 2050 (2, 4). The impact of the high prevalence of dementia in the elderly is noteworthy, as seen in the substantial direct healthcare costs as well as in the devastating social costs for individuals and their families and caregivers (2). Yet, despite the growing importance of understanding dementia, we are still in search of effective methods for its diagnosis and treatment.

In this review, we provide a summary of the potential role of the amino acid D-serine, a potent co-agonist at the N-methyl-D-aspartate glutamate receptor (NMDAR), in normal and pathological aging, with a focus on neurocognition. A brief discussion on the diagnostic and therapeutic potential of D-serine is also included. The evidence suggests that this is a promising avenue of research into the pathophysiology of neurocognition and its potential treatment in dementing illnesses. Literature searches were performed in PubMed and Web of Science for the period January 1970 to May 2021, and the key search terms used were “D-serine and dementia”, “D-serine and Alzheimer's disease”, “D-serine and mild cognitive impairment”, “D-serine and LTP”, as well as “D-serine and NMDA receptors”. Only papers in English were used in preparation of the review, and some of the review papers found were searched for additional relevant references. Each reference used was screened by at least two of the authors.

## Physiology of Normal Aging

Aging is a normal dynamic process, characterized by the development of a mild inflammatory environment and a progressive deterioration of certain physiological functions, including in the central nervous system (CNS) (5, 6). Although cognitive decline is relatively common in old age, the relationship between aging and degenerative dementias such as AD remains unclear. Whereas aging is a risk factor for AD, it is not inevitable that AD be part of the aging process. While obvious and oftentimes widespread structural changes can be seen within the CNS with dementia pathophysiology, normal aging is not associated with a significant loss of neurons (7); rather, brain alterations in normal aging are much more subtle, involving changes in connectivity and altered functions at the cellular and molecular level (8). Several cognitive domains are affected in normal aging and dementia, including learning and memory (particularly for newly acquired information), processing speed, working memory, and executive function (9, 10). An intriguing feature of aging is the variation of degree of cognitive impairment between individuals, from a mild deficit to a severe dementia, as in the case of AD (11, 12).

The decline in learning and memory performance during non-pathological aging appears to be primarily the result of alterations in neuronal network plasticity within the hippocampus (12). Memory formation is viewed as being closely dependent on the capacity of the brain to regulate long-lasting changes in neuronal communication *via* synapses, and appears to be proportional to the strength of those communications (13, 14). The first convincing support for neuronal plasticity changes underlying changes in cognition came in the 1970s when long-term potentiation (LTP), a mechanism now known to underpin synaptic strengthening critical for learning and memory, was characterized in the hippocampus (15). It was later shown that LTP was regulated in large part by NMDAR signaling (16–18).

Dynamic synapses facilitate remodeling of neuronal circuits, and changes in the functional properties of these networks could play a critical role in the induction of age-related memory decline (19). However, the mechanisms governing dynamic synapses in the brain are still not well understood (20, 21). The hippocampus is the area most frequently implicated in memory decline and this structure seems to be particularly vulnerable to aging (22–24). Interestingly, the circuits that are vulnerable to aging are composed to a large extent of glutamatergic neurons (25).

Proper brain functioning requires healthy neurons and neuronal connections, which in turn require properly functioning neurotransmitters and enzymes that supply these dendritic and neuronal connections. It has been shown repeatedly that deficits in glutamatergic transmission mediated by the NMDAR are related to cognitive impairment in both laboratory animals and humans. Administration of an NMDAR antagonist in rhesus monkeys impairs recognition memory (26), which represents cognitive impairment (27). Similarly, specific ablation of *GRINs* (Glutamate Ionotropic Receptor NMDA Type 1-3), i.e., the genes that encode for subunits of the NMDAR heterotetrameric complex, in the hippocampus or pharmacological blockade of NMDAR function can lead to brain atrophy, impaired neuroplasticity, reduced LTP and deficits in learning and contextual memory (18, 28, 29). In contrast, increasing NMDAR function by over-expression or reduced degradation in the hippocampus can enhance LTP and learning (30, 31).

Particular attention has been paid to learning and memory, and to whether activation of NMDARs could be altered in the course of aging. Various studies in wild-type rodents have revealed that aging is associated with reductions in the magnitude of LTP in the hippocampus and have implicated alterations in NMDAR signaling and a decline in the activation of NMDARs associated with a decrease in levels of D-serine, a co-agonist at the NMDA receptor. Therefore, age-related decreases in D-serine could be contributing to the cognitive decline (10). Since activation of the NMDAR co-agonist-binding site by D-serine and glycine is mandatory for the induction of synaptic plasticity, the LTP rescue observed in aged animals after supplementation with the co-agonist D-serine also suggests that the mechanisms managed by endogenous D-serine are altered with age (11).

## D-Serine Physiology, Metabolism and Role in Aging

**Figure 1:**
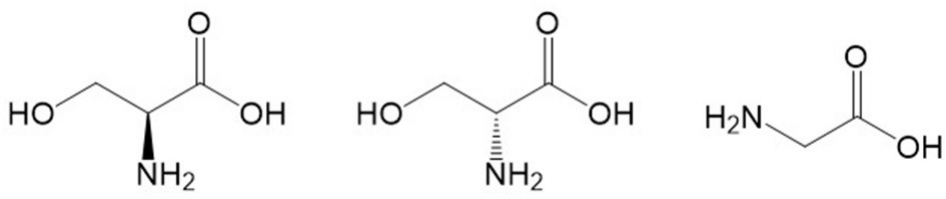
Chemical structures of L-serine (left), D-serine (center) and glycine (right). Structures were located with Google and drawn with ChemDraw.

Memory formation relies on the capacity of neuronal networks to manage long-term changes in synaptic communication. This property is driven, at least in part, by NMDARs (32). The NMDAR is a tetrameric ion channel that may be composed of many configurations of three subunits, i.e., GluN1, GluN2, and less commonly, GluN3 (33–35). To be activated, the NMDAR requires simultaneous binding of the agonist glutamate to the GluN2 subunit and a co-agonist to GluN1 (34–37). This binding is crucial for NMDAR activation and originally it was thought that the major co-agonist was glycine (10, 36, 37); however, later studies found that D-serine is more potent than glycine at binding to the co-agonist site on the GluN1 subunit of the NMDAR and stimulating the receptor in forebrain regions, including hippocampus (38). D-Serine has a regional distribution in the brain more similar to that of NMDARs than does glycine (39–41) and it has been reported that D-serine acts primarily at synaptic NMDARs whereas glycine acts primarily at extrasynaptic NMDARs (38). Interestingly, glycine is similar structurally to D-serine (Figure 1) and it is formed by conversion of L-serine catalyzed by the enzyme serine hydroxymethyltransferase.

Balanced NMDAR activity is required for optimal brain function. Hypo- or hyper-function of NMDAR-mediated neurotransmission can result in cognitive dysfunction or neurotoxicity, respectively. Depletion of D-serine diminishes NMDAR activity, LTP, and synaptic plasticity (33). NMDAR-mediated neurotransmission and its modulation by D-serine play a critical role in memory formation, learning, and neuronal plasticity (34, 42–44). In CNS development, D-serine shapes synaptogenesis and neuronal circuitry through activation of NMDARs and it is also a key player in astrocyte-mediated LTP associated with hippocampal plasticity (20).

The reports by Hashimoto et al. were the first to demonstrate high concentrations of D-serine in the rodent brain and in the human brain (45, 46). It was only later discovered that D-serine is enriched in brain regions containing high concentrations of NMDARs, such as the cerebral cortex, hippocampus, and amygdala (41). The source of D-amino acids in mammals was historically attributed to diet or intestinal bacteria (47) until the racemization of L-serine by serine racemase was identified as the endogenous source of D-serine (48) (see Figure 1 for structures of L- and D-serine). Serine racemase was first described to be exclusively present in astrocytes (49–51), but subsequent work has shown that serine racemase is also present in neurons (52). Thus, D-serine may be a glial transmitter as well as a neurotransmitter, and this has been a matter of considerable controversy [for discussions of this matter see: (52–54)]. Wolosker et al. (52) proposed that L-serine is synthesized in astrocytes and then shuttled to neurons where it is converted to D-serine. For a detailed description of D-serine circuits and the “serine shuttle”, see Wolosker and Balu (55).

Serine racemase is expressed by many CNS cells, including pyramidal neurons in the cerebral cortex and the CA1 region of the hippocampus (41, 56), regions that also have high levels of D-serine (57). Wong et al. (58) have shown an age-dependent dendritic and postsynaptic localization of serine racemase in CA1 pyramidal neurons of the mouse. These same researchers, in studies using serine racemase knockout (KO) mice, showed a cell-autonomous role for this enzyme in regulating synaptic NMDAR function at Schaffer collateral (CA3)-CA1 synapses and found that single-neuron genetic deletion of serine racemase eliminated LTP at the age of 1 month and that this loss of LTP could be rescued by administering D-serine (58). The enzyme responsible for the catabolism (breakdown) of D-serine is D-amino acid oxidase (DAAO); this enzyme is most abundant in cerebellum and brain stem, areas with low levels of D-serine (59).

D-Serine levels vary across different CNS areas. The level of D-serine is in the order of 200–300 pmoles per milligram of tissue in the hippocampus and frontal cortex in mice, 20-fold higher than in the pancreas, lung, or testis and almost 50-fold higher than in muscle (60). Within the brain, highest levels of D-serine are in the cortex and hippocampus, and there are much lower levels in the cerebellum and brain stem, likely reflecting the regional variation in expression of serine racemase and DAAO (review: 61).

D-Serine, through its regulatory effect on glutamatergic transmission, participates in multiple processes, including synaptic plasticity (61, 62), cell migration and synaptogenesis (41, 63), and in homeostatic functions, as a mediator of hypercapnia-induced respiratory response (64). The production of D-serine and its tightly regulated release, mainly through calcium-dependent exocytosis (65), keep its concentration within a narrow range. Any deviation from this range may lead to pathology, with abnormally increased levels of D-serine associated with NMDAR-mediated neurotoxicity (66–68) and abnormally decreased levels of D-serine associated with impairments in functional plasticity and with memory deficits (11). The complexity of its actions and its modulatory effects are not well understood; indeed, Coyle et al. (69) referred to D-serine as a “shape-shifting NMDAR co-agonist” and provided a possible explanation for these dueling effects of D-serine on driving neuronal plasticity or neurodegeneration based on the localization of the activated NMDARs involved. It is known that synaptic NMDARs prompt trophic effects while extra-synaptic NMDARs on the dendrites or soma drive excitotoxicity (38, 70, 71). Coyle et al. (69) propose that D-serine synthesized by serine racemase binds preferentially to synaptic NMDARs and facilitates glutamatergic neurotransmission, while proliferation of inflammatory A1 astrocytes results in a new source of D-serine that is released into the extracellular space to activate extra-synaptic NMDARs.

D-Serine levels in the CNS change during development and aging. In early developmental stages, a transient increase in D-serine production matches a transient increase of NMDAR activity (72). The early postnatal period with high D-serine levels in glia coincides with a period of intense plasticity, synaptogenesis and maturation in the CNS, suggesting the existence of distinct functional roles for D-serine throughout development (72). Healthy newborn children have elevated CSF D-serine levels that are rapidly reduced during the first year of life and reach 15% of the initial concentration at 3 years of age (73).

In the hippocampus of normal aged rats, both D-serine (but not glycine) and serine racemase levels are decreased relative to younger rats (74, 75). In contrast, these reductions in D-serine and serum racemase are not observed in the LOU/c/jall rat strain regardless of age (5, 76). The LOU/c/jall strain of rat (derived from the Wistar strain) is a model of healthy aging (with resistance to obesity and lower oxidative metabolic rates than the routinely used other inbred strains of rats) (76). Interestingly, the possibility that D-serine-related pathways could be targeted by the age-related accumulation of reactive oxygen species (ROS) has been suggested (5), and LOU/c/jall rats do not develop oxidative stress (5, 76).

## D-Serine, NMDARs and Cognitive Impairment in AD/Dementia

### Animal Studies

Characterizing the processes associated with hippocampal dysfunction has been an area of focus in research on AD, where β-amyloid (Aβ) deposits, intracellular neurofibrillary tangles, abnormal tau protein phosphorylation and synaptic loss are typical pathological features (77–79). The pathological changes that are detected in the brains of patients with AD, such as the presence of amyloid plaques and neurofibrillary tangles, are now known to appear several years before the development of clinical symptoms. As such, current research is focusing more on early detection and treatment in these earlier stages in the hope of delaying the onset or slowing the progression of AD.

Although NMDAR function is vital for memory and cognitive function, its role in the pathophysiology of AD is still not completely understood. NMDAR over-activation can lead to cell death mediated by calcium overload. The associated excitotoxicity is one of the accepted neurochemical models of AD in rodents and may be involved with the pathophysiology associated with Aβ, a hallmark of the pathogenesis of AD (80–82). Interestingly, different forms of Aβ aggregates increase glutamate release from neurons and astrocytes (2, 83) and Aβ can increase NMDAR activity and induce inward Ca^2+^ current and neurotoxicity; this NMDAR activation may stimulate Aβ production and Aβ-associated synaptic loss (2). Aβ deposition appears to play an important role in the pathophysiology of AD, and the mechanism underlying glutamate excitotoxicity in AD may be related to Aβ deposition (84, 85). Aβ aggregation interferes with NMDAR-mediated neurotransmission, suppressing NMDAR-dependent synaptic function and LTP, which may lead to cognitive impairment (86–89). Furthermore, Aβ can lead to intracellular trapping of NMDARs, decreasing LTP; this effect can be rescued by a Reelin- and Src kinase-dependent tyrosine phosphorylation in the GluN2 subunit of the NMDARs, restoring normal synaptic plasticity (90). In addition to Aβ, apolipoprotein E4 (APOE4), a protein isoform that has lower Aβ-binding capacity than APOE2 and APOE3, and is a genetic risk factor for AD (91), reduces NMDAR function and synaptic plasticity by impairing APOE receptor recycling (92).

Aβ peptides have also been shown to stimulate the synthesis and release of D-serine (93) in preclinical models (80). The excessive D-serine release from neurons and glia leads to synaptic loss and stimulation of extra-synaptic NMDAR currents (94, 95). Excessive levels of D-serine create a dramatic overload of Ca^2+^ (96), and degradation of D-serine by DAAO or D-serine deaminase protects against cell death (97). Dysfunctional D-serine metabolism may be a downstream outcome of Aβ toxicity, and excess D-serine release may contribute to neuronal death in AD through excitotoxicity. However, whether levels of free D-serine are elevated in the brains of AD is still a matter of debate as levels vary depending on brain region and stages of pathology (10).

Ongoing interest in amyloid precursor protein (APP), the precursor of the Aβ peptide in AD, has been refueled by evidence indicating its multifaceted complex role in synaptic (patho)physiology and development (98). Animal studies have shown that a lack of APP impairs the structural plasticity of dendritic spines (important for cognition and memory) and that APP plays a key role in regulating D-serine homeostasis, which is an important factor in synaptic plasticity in the adult brain (98). These authors measured cortical extracellular and total D-serine concentrations in APP-KO mice and found an increase in concentrations of total D-serine, but a concurrent decrease in concentrations of extracellular D-serine. Treatment with exogenous D-serine not only restored the extracellular D-serine levels and synaptic plasticity, but also normalized the concentrations of total D-serine and rescued the cognitive deficit observed in the APP-KO mice. These results suggest that the maintenance of D-serine homeostasis requires APP and demonstrate D-serine's essential role in adaptive remodeling in the adult brain (98).

Microglia are the main immune effector cells of the brain and the main source of inflammatory cytokines and reactive oxygen species (ROS) in the CNS (5). Alterations in the activation and regulation of microglia can promote a chronic inflammatory condition in the CNS in normal and pathological aging (5), an inflammatory environment termed immunosenescence. This process induces changes in gene expression related to the immune response and inflammation, causing increased susceptibility to inflammatory responses to stressors, which could facilitate the onset of neurodegeneration (5, 6, 99–102). Activation of microglial cells, as part of a chronic inflammatory response, is a prominent component of AD that drives neurotoxicity through the release of excitotoxins including glutamate, and increased activity of Aβ, which not only promotes glutamate release from microglia, but also stimulates expression of serine racemase and D-serine release from these glial cells (2, 93, 103). Aβ also promotes serine racemase activity through increases in intracellular levels of calcium, upregulating the activity of the enzyme. How much of the changes in D-serine levels during aging are determined by microglial cell actions is unclear. However, it is speculated that age-dependent changes in microglia regulation result in neuroinflammation and increased oxidative stress (104), in turn eventually activating production of D-serine by glia and neurons in AD (5).

The functioning of neuronal networks within the CNS requires high levels of oxygen, and the CNS is particularly sensitive to oxidative stress (105). Studies have found that antioxidant levels in the brain are low compared to other organs (106). Changes in redox regulation in the CNS may be accompanied by neuronal dysfunction, particularly alterations of synaptic plasticity (107, 108). Assuming synaptic plasticity is an essential neuronal mechanism for learning and memory (13, 14), it may be a preferred target by which oxidative stress could alter memory functions. DAAO plays a key role in the process of oxidative stress and results in formation of ROS; through this effect and its regulatory function on NMDARs by reducing levels of D-serine, DAAO may play an important role in the process of aging and age-related cognitive decline (109). Nagy et al. (110) studied the effects of the DAAO inhibitor CPD30 on passive avoidance learning and neuronal firing activity in rats and concluded that inhibition of DAAO is an effective strategy for cognitive enhancement; CPD30 increased hippocampal firing and reversed MK-801-induced memory impairment in the passive avoidance test.

### Human Studies

The preclinical studies mentioned above have suggested that while normal aging may result in decreases in D-serine synthesis and levels, NMDAR activity, the magnitude of LTP and synaptic plasticity (all of which may be reversed by administration of D-serine), pathological aging may involve activation of serine racemase, increased levels of D-serine, NMDAR hyperstimulation and excitotoxicity, resulting in dementia (Table 1).

**Table 1:** Abnormal D-serine function in normal aging and Alzheimer's disease.

	**Serine** **racemase** **expression**	**D-serine** **levels**	**NMDARs**	**Cognitive** **changes**
Normal Aging	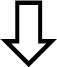	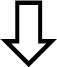	Down-regulation, leading to reduced LTP and impaired synaptic plasticity	Variable learning and memory deficits
AD	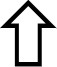	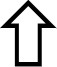	Over-stimulation, interactions with activated microglia and Aβ, increased release of glutamate, excitotoxicity	Dementia

*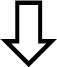 = decrease; 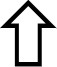 = increase; AD, Alzheimer's disease; NMDARs, N-methyl-D-aspartate receptors; LTP, long-term potentiation; Aβ, β-amyloid. [adapted from Billard (11)]*.

Madeira et al. (16) conducted a comprehensive combined clinical-preclinical study on D-serine in AD. D-Serine levels were measured in post-mortem hippocampal and cortical samples from non-demented individuals and AD patients. D-Serine was also measured in hippocampus from wild type rats and mice after intracerebroventricular injections of Aβ and in the APP/PS-1 transgenic mouse model of AD. In addition, D-serine levels in CSF of people with probable AD were also measured and compared to those of patients with normal pressure hydrocephalus or major depression, and to healthy controls. D-Serine levels were higher in the post-mortem hippocampus and parietal cortex samples of AD patients than in healthy controls. The researchers also found higher levels of D-serine and serine racemase in all the rodent models compared to controls. Furthermore, D-serine levels were higher in the CSF of probable AD patients compared to the non-demented control groups; mean D-serine levels in the probable AD group were five-fold higher than in healthy controls, and approximately two-fold higher than in the depression or hydrocephalus groups. These researchers concluded that D-serine levels in brain and CSF are increased in AD and that D-serine might be a candidate for early AD diagnosis (16). In contrast, three earlier studies using post-mortem prefrontal, parietal, frontal or temporal cortical tissue failed to detect altered D-serine levels between AD and controls (111–113). All of the post-mortem studies had small sample sizes and a wide range of participant ages and postmortem collection times. One study (16) had equal numbers of males and females, one (113) had all male participants and the other two studies (111, 112) did not indicate the male/female ratio.

## Potential Role of D-Serine in Diagnosis of AD

Significant efforts are being made to identify diagnostic markers and modifiable risk factors for AD, specifically any factor that influences the earliest stages of the disease process, when intervention might still provide therapeutic benefit. In this context, CSF levels of Aβ, total tau protein and hyperphosphorylated tau (p-tau) have now been included in diagnostic guidelines (114). Such CSF biomarkers have been advocated for research purposes, but sensitivity and specificity issues have generally raised concerns about their widespread clinical use (15). Madeira et al. (16) proposed that combining CSF D-serine levels with the Aβ/tau index could markedly increase the sensitivity and specificity of diagnosis of probable AD. However, Biemans et al. (115) and Nuzzo et al. (116) did not find a difference in CSF D-serine levels between AD patients and elderly controls.

Lin et al. (109) found increased levels of DAAO in the serum of patients with mild cognitive impairment (MCI) and AD and observed that the severity of cognitive deficits correlated positively with DAAO blood levels, suggesting that this enzyme catabolizing D-serine may also serve as a biomarker for MCI/AD. These researchers found that DAAO levels were significantly lower in healthy controls than in the patients, and moreover, lower in patients with amnestic MCI than in those with moderate to severe AD (109). In the same study, D-serine levels in serum were reported to be higher in AD patients than in the healthy controls. The clinical benefit of DAAO inhibition in AD may be mediated in part by an antioxidant effect since D-serine degradation by DAAO generates hydrogen peroxide, a precursor to many ROS (10, 109). In a later study of D-serine levels in 144 patients with varying degrees of cognitive impairment, Lin et al. (117) concluded that higher D-serine levels predict worse cognitive function, particularly with regard to word recall, orientation, comprehension, and word-finding.

In a recent metabolomics study in a cohort of women aged 65–80 years old, Kimura et al. (118) reported a higher D-proline/(D-proline+L-proline) ratio in women with MCI compared to matched controls, and found this biomarker's accuracy was improved by further adding the D-serine/(D-serine+L-serine) ratio. Piubelli et al. (119) measured serum levels of D- and L-serine in AD patients with either a score of 1 (mild dementia) or 2 (moderate dementia) in the Clinical Dementia Rating Scale, and found that D-serine levels and the D-serine/total serine ratio increased significantly with disease progression. These researchers suggested using the combination of the above ratio with other blood-based biomarkers presently under development and reviewed by Hampel et al. (120).

The role of D-serine in AD is complex and the literature is often ambiguous. It has been suggested that some of the differences between findings in laboratory animals and human AD patients could be due to the fact that current animal models do not mimic the slow progression and the changes in Aβ and tau protein that occur in AD in humans (11). It has also been proposed that studies on D-serine and AD should be done at various stages of AD since at early stages with low levels of Aβ oligomers there is also decreased synthesis of L-serine and, hence, decreased D-serine levels and weaker NMDAR activation. However, at later stages when there is increased soluble Aβ, glia start to express more serine racemase and release large amounts of D-serine, resulting in NMDAR over-activation and resultant excitotoxicity, neurodegeneration and marked memory deficits (117). There is also some speculation that D-serine increases observed in AD patients may be part of a protective mechanism to counter Aβ signaling and prevent AD pathology (10).

## Treatment Potential of D-Serine

As mentioned above, there is a loss of production of D-serine and a decline in NMDAR activation and a corresponding reduction of LTP magnitude in the normal aging process, which can be reversed in animal models by administration of D-serine (11). These findings imply that increasing D-serine levels in cases of initial cognitive decline or in early stages of AD may be therapeutically useful (10).

Findings that the co-agonist modulatory site was not saturated *in vivo* prompted investigators to consider whether exogenous D-serine could act as a cognitive enhancer (10). Although the focus of the present review is on dementia, it should be mentioned that much of the research on the effects of D-serine in cognition in humans has been done on schizophrenia (57, 121–130), reporting either cognitive benefits (121, 122, 125, 126, 130) or no effects on cognition (123, 128, 129). It is difficult to compare the studies since they were performed at several doses, the patients were taking antipsychotics (which presents a possible confound), and a variety of tests were conducted to measure cognition. Most of the studies were carried out using a daily dose of 30 mg/kg, but Kantrowitz et al. (126, 130) also used higher doses (60 and 120 mg/kg) and reported improvements in cognition.

**Figure 2:**
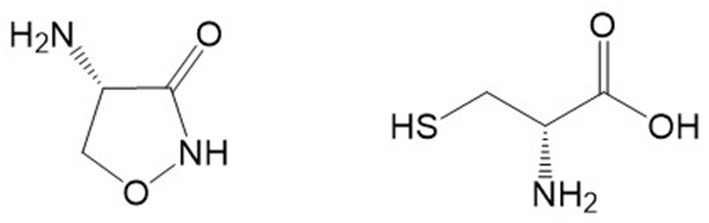
Chemical structures of D-cycloserine (left) and D-cysteine (right). Structures were located with Google and drawn with ChemDraw.

D-Serine administration can improve cognition in aged rodents and correct age-related decline in synaptic plasticity (10). In mouse models, the learning deficits caused by NMDAR hypofunction can be rescued by administration of D-serine (131). Although conflicting results have been reported, D-cycloserine (Figure 2; a cyclized form of D-serine that is hydrolyzed to give D-serine and hydroxylamine) has been reported to improve memory functions in animal studies and in dementia patients (132, 133). Lin and Lane (133) speculated that D-cycloserine may have different effects on mood and learning depending on the stage of dementia involved. D-Serine given intraperitoneally to rats can increase NMDAR activation in the hippocampus and improve social memory in rats and recognition and working memory in mice (10). The potency of exogenous D-serine to enhance NMDAR activation appears significantly higher in hippocampal slices from aged rats when compared to effects in younger adult rats (134). Nikseresht et al. (135), using a rat model of AD (intracerebroventricular injection of Aβ), reported a synergistic memory-enhancing effect of D-serine and the mitochondrial calcium uniporter blocker RU360. The findings in this report suggested that the coadministration of these drugs ameliorated memory impairment, probably in part through an increase in hippocampal levels of cyclic AMP response element binding protein (CREB) and brain-derived neurotrophic factor (BDNF).

In a randomized controlled clinical trial (RCT) by Avellar et al. (9), 50 healthy elderly human adults received a single dose of D-serine or placebo, and the effects of D-serine administration on cognitive test performance and a mood scale were measured. In addition, blood samples were analyzed for levels of D-serine, L-serine, glutamate and glutamine. D-Serine levels measured while the participants were on placebo were inversely associated with aging. D-Serine administration improved performance in the Groton Maze Learning Test of spatial memory, learning and problem solving. Individuals who achieved higher increases in plasma D-serine levels after administration improved more in test performance. D-Serine administration was not associated with any significant changes in other cognitive domains, such as verbal working memory, visual attention or cognitive flexibility. There were also no changes observed in mood (9). In a similar study, but in young healthy adults, Levin et al. (136) demonstrated that D-serine administration improved attention, verbal learning and memory as well as subjective feelings of sadness and anxiety.

These above studies suggest an important role for D-serine in brain networks underlying memory impairment and provide useful information in the search for new therapeutic strategies for the treatment of memory deficits. However, an important question is whether the improvements seen so far with the addition of D-serine in animal models and healthy human controls will have real-life effects in AD (11).

## Other Treatment Approaches Related to D-Serine

In the aging brain, ROS accumulation may trigger age-related reduction of cognitive function through oxidative stress. Consequently, ROS accumulation could be viewed as a major process acting on the D-serine-related pathway in the aging hippocampus, especially considering that serine racemase activity is particularly sensitive to oxidative stress (105). Long-term dietary supplementation with L-N-acetylcysteine (L-NAC, a precursor to the antioxidant glutathione) prevented oxidative damage in the hippocampus and restored D-serine-dependent NMDAR activation and LTP induction in aged rats (20). These data provide evidence that maintaining elevated D-serine levels in the aging hippocampus through the control of the redox state is able to prevent the cellular injury underlying cognitive aging, specifically in the CA1 hippocampal area (11).

An increase in D-serine availability in the brain could be achieved by reducing its degradation by DAAO. Treatment of rats with a DAAO inhibitor has been reported to increase levels of D-serine in the cerebral cortex and midbrain (137). Although DAAO KO mice have been reported to have markedly increased levels of D-serine in cerebellum and brain stem but little or no change in D-serine levels in cortex or hippocampus (138, 139), support for a physiological role for DAAO in modulating cognition comes from the enhanced learning abilities reported for DAAO KO mice (57, 140). The DAAO inhibitor sodium benzoate, which also modulates the immune system and is an antioxidant, has been shown to improve cognition, global functioning and positive and negative symptoms of schizophrenia (141). Modi et al. (142), using an animal model of AD, reported that sodium benzoate reduced oxidative stress and protected memory and learning. In addition, in RCTs of 6 weeks daily treatment with sodium benzoate, Lin and colleagues reported that cognitive scores were improved in early stage dementia patients and in women, but not men, with later phase dementia (143).

The D-amino acid D-cysteine, which is derived from the gut, and is structurally related to D-serine (it is also referred to as thioserine; Figure 2) also exerts neuroprotection, but it does so *via* a DAAO-dependent conversion to H_2_S (144). Interestingly DAAO has greater affinity for D-cysteine even though D-serine is found in far greater concentrations in the brain (145). It is all the more interesting that D-cysteine has been shown to be a potent inhibitor of serine racemase (146), thereby making it a potential treatment for pathologies where D-serine might exert deleterious effects, such as in AD.

## Limitations in the Use of D-Serine as a Biomarker and Treatment

The fact that body fluid levels of D-serine have been reported to be altered in other psychiatric and neurological disorders, such as depression, anxiety, schizophrenia, bipolar disorder and hydrocephalus (16, 61, 147, 148) suggests that D-serine would not be a specific biomarker for AD. There are also potential challenges for the clinical use of D-serine, including the possibility of nephrotoxicity (149, 150). However, this nephrotoxicity may only be a problem with rats since it has not been reported in other species, including rodents such as mice and rabbits (151, 152). Even in rats, the nephrotoxicity is reversible and appears to occur only at high doses (152). In a comprehensive review of safety of D-serine across species, Meftah et al. (152) listed the studies on humans with D-serine that have been published and reported that only one subject in one study showed renal abnormalities. These researchers concluded that D-serine is safe and well tolerated in humans even at the highest dose (120 mg/kg) tested to date, but that people with pre-existing renal dysfunction should be excluded from clinical studies. Co-administration of a DAAO inhibitor with D-serine may be a strategy to prevent nephrotoxicity since lower doses of D-serine could be used and hence formation of peripheral metabolites of D-serine reduced (153). In mice, treatment with a DAAO inhibitor has been reported to render a low dose of D-serine effective in treating pre-pulse inhibition deficits caused by the NMDAR antagonist dizocilpine, compared to the same dose of D-serine alone (154).

Poor oral bioavailability can also limit the effects of D-serine on cognition. Accordingly, D-serine had better effects on cognition when administered as an adjunct to patients with schizophrenia when higher doses such as 60 mg/kg/day or higher were used (review: 61). In general, poor oral D-serine bioavailability may account for mixed results in clinical trials, and alternative treatment paradigms may need to be considered, including larger doses of D-serine or a combination of D-serine and sodium benzoate (thus using lower doses of both drugs while retaining high efficacy). Because D-serine and sodium benzoate have different pharmacokinetic and pharmacodynamic profiles, it is possible that D-serine may be especially useful for treating depression because of its acute and chronic antidepressant effects, whereas sodium benzoate may be a safer approach in older adults with impaired renal function (10).

## Challenges and Possible Future Directions in Research on D-Serine and Cognition

Considerable evidence in the literature supports the involvement of D-serine in reduction of cognitive deficits, but there are some contradictory findings that indicate that further research is warranted. For example, Capitao et al. (155), in a study of a single dose (60 mg/kg) in human volunteers, found that D-alanine modulated emotional processing while D-serine did not. Some researchers have questioned the physiological role of DAAO in controlling D-serine availability because this enzyme is expressed at low levels in forebrain areas relevant to cognition such as the hippocampus and cortex, and D-serine levels have been reported to be elevated markedly in the cerebellum and brain stem but not in cortex or hippocampus of DAAO KO mice (138, 139). However, other researchers have found that systemic administration of a DAAO inhibitor to rats increases levels of D-serine in the cortex (137). Labrie et al. (140) reported that DAAO KO mice had a marked increase in levels of D-serine in the cerebellum, but also had a relatively small, but significant, increase in D-serine levels in the hippocampus and showed enhanced extinction and reversal learning.

Although it has been proposed that CSF and/or serum levels of D-serine could be novel biomarkers for AD (16, 119, 156), other researchers have reported that D-serine levels in these body fluids are unaltered in AD (115, 116). It has also been reported that perinatal epigenetic mechanisms play a role in the regulation of levels of D-serine in the brain (157), and future studies in AD should include epigenetic investigations on expression of serine racemase and DAAO genes. Dysregulation of aerobic glycolysis in the brain is often observed early in the course of AD, and Le Douce et al. (158) have shown that the astrocytic biosynthetic pathway for L-serine (the precursor for D-serine), which branches from glycolysis, is impaired in young AD mice and in AD patients. These researchers found that dietary supplementation with L-serine prevented the synaptic and behavioral deficits in AD mice, which suggests that oral L-serine could be a therapy for AD.

## Relevance of D-Serine to Comorbid Depression, Anxiety and Other Behavioral Changes in Dementia

The focus of this review has been on the involvement of D-serine in cognitive deficits, but dementia is complex and often there is a high degree of comorbidity with depression, anxiety, aggression, and/or sleep disorders. There is now an extensive body of literature indicating involvement of D-serine in each of these disorders. It may seem contradictory for D-serine to have antidepressant effects considering the known antidepressant effects of the NMDAR antagonist ketamine (159), but several preclinical and clinical studies report antidepressant actions of D-serine [reviews: (61, 160, 161)]. It has been proposed that the antidepressant actions of ketamine and D-serine may be due to common effects on α-amino-3-hydroxy-5-methyl-4-isoxazole propionic acid (AMPA) glutamate receptors and similar differential actions on synaptic *vs*. extra-synaptic NMDARs (160). Wolosker and Balu (55) have provided a comprehensive review of mainly preclinical studies suggesting a role of D-serine in fear conditioning and anxiety disorders. As an abnormal social behavior, aggression (often studied in mice as social interaction deficits with intruder strains of mice) has been observed in rodents to show an association with NMDAR function (162–165). Both D-cycloserine and D-serine have been reported to improve impaired social interaction skills, for example in inbred Balb/c mice used as models for autism (164–167). Nagai et al. (168) reported that mice treated neonatally with polyI:C (elicits viral-like immune responses) had emotional and cognitive deficits which could be ameliorated in adulthood by treatment with D-serine. With regard to sleep disorders, studies in mammals and Drosophila flies have shown that NMDARs and D-serine participate in sleep regulation (169–171). Drosophila has been used as a model for genetic studies of sleep for several years (172). In a detailed study of sleep in this model, Dai et al. (173) showed that sleep is regulated by D-serine through NMDAR1 and that intestinal expression of serine racemase is important for this sleep regulation.

Longitudinal studies, both preclinical and clinical, involving larger samples sizes will be needed in future research on D-serine, and such investigations should include both males and females, along with assessments of the comorbid disorders mentioned above.

## Summary

In normal aging there is development of a mild inflammatory environment and progressive deterioration of several physiological functions, including cognition involving learning and memory performance. With aging, the degree of cognitive impairment can vary markedly among individuals. Memory formation depends on the capacity of the brain to regulate long-lasting changes in neuronal communication *via* synapses, and these changes in neuronal plasticity are dependent on LTP, which is regulated in large part by NMDARs. Functioning of NMDARs is in turn dependent on co-agonists, the most important of which appears to be D-serine. Numerous animal studies have shown that even with normal aging there is a reduction in the magnitude of LTP in the hippocampus accompanied by a decline in NMDAR action and a decrease in production and levels of D-serine. It has also been demonstrated in animal models that administration of D-serine can rescue the reduced NMDAR function and loss of LTP observed in aging.

Preclinical studies suggest that D-serine may be useful in treating cognitive impairment, but while abnormally decreased levels of D-serine are associated with impairments in functional plasticity, abnormally increased levels of D-serine can be associated with NMDAR-mediated excitotoxicity such as occurs in later-stage AD. Activation of microglia is part of a chronic inflammatory response in AD that increases release of glutamate and D-serine from glia and neurons, and Aβ also stimulates expression of serine racemase in microglia. It has been suggested that with cognitive deficits associated with normal aging and in early AD, there may be decreased expression of serine racemase, decreased levels of D-serine, NMDAR down-regulation and impaired synaptic plasticity, while in advanced AD serine racemase activation and D-serine levels are increased and NMDARs are overstimulated, resulting in excitotoxicity and dementia.

D-Serine and DAAO have been proposed as possible biomarkers in the diagnosis of AD, although there have been conflicting results reported and differences found in animal models and humans. Current animal models do not mimic the slow progression and the changes in Aβ and tau protein that occur in humans; it has also been proposed that future studies on D-serine in humans should be done at several stages of AD. Research to date suggests that earlier stages of AD would benefit from D-serine supplementation, whereas D-serine supplementation should be avoided in later stages of AD. DAAO inhibitors may also be useful for increasing brain D-serine levels and enhancing learning.

Although we understand a great deal about the roles of D-serine in brain function, about changes in its brain levels with normal and pathological aging, and about its potential role as a cognitive enhancer from experimental and preclinical studies, much still remains to be learned about its potentially targetable role in development, treatment and possibly even prevention of dementia in a clinical setting.

## Author Contributions

MO and GB conducted the initial literature search. Each reference was screened by at least two of the authors. MO prepared the initial draft of the manuscript and all authors then contributed to editing. All authors have agreed to submission of this version of the manuscript.

## Funding

DM acknowledges financial support from the Office of Research, College of Medicine, University of Saskatchewan as well as from a philanthropic Saskatchewan family. DM also acknowledges the *Saskatchewan Research Chair in Alzheimer disease and related dementias* funded jointly by the Alzheimer Society of Saskatchewan and the Saskatchewan Health Research Foundation. GB has a TRIP Research Allowance (TRP-GB) from the Faculty of Medicine & Dentistry at the University of Alberta. EF acknowledges funding from CIHR (FRN 201803).

## Conflict of Interest

GB is an advisor to NeuraWell Therapeutics. The company had no involvement with this review. The remaining authors declare that the research was conducted in the absence of any commercial or financial relationships that could be construed as a potential conflict of interest.

## Publisher's Note

All claims expressed in this article are solely those of the authors and do not necessarily represent those of their affiliated organizations, or those of the publisher, the editors and the reviewers. Any product that may be evaluated in this article, or claim that may be made by its manufacturer, is not guaranteed or endorsed by the publisher.
